# Differential Octopaminergic Modulation of Olfactory Receptor Neuron Responses to Sex Pheromones in *Heliothis virescens*

**DOI:** 10.1371/journal.pone.0143179

**Published:** 2015-12-09

**Authors:** N. Kirk Hillier, Rhys M. B. Kavanagh

**Affiliations:** Department of Biology, Acadia University, 33 Westwood Avenue, Wolfville, Nova Scotia, B4P 2R6, Canada; University of Houston, UNITED STATES

## Abstract

Octopamine is an important neuromodulator of neural function in invertebrates. Octopamine increases male moth sensitivity to female sex pheromones, however, relatively little is known as to the role of octopamine in the female olfactory system, nor its possible effects on the reception of non-pheromone odorants. The purpose of this study was to determine relative effects of octopamine on the sensitivity of the peripheral olfactory system in male and female *Heliothis virescens*. Single sensillum recording was conducted in both sexes following injection with octopamine or Ringer solution, and during odorant stimulation with conspecific female sex pheromone or host plant volatiles. Results indicate that octopamine plays a significant modulatory role in female sex pheromone detection in female moths; and that male and female pheromone detection neurons share distinct pharmacological and physiological similarities in *H*. *virescens* despite sexual dimorphism at the antennal level.

## Introduction

The olfactory system is important for sensory detection and is particularly important for invertebrates [1]. Insects use olfaction to engage in critical behaviors, including selection of food, choice of oviposition sites, identification of predators, and detection and selection of conspecific mates [1]. In general, there are two main classifications of odors important to phytophagous insects: host plant volatiles, and pheromones, being important for oviposition/feeding and mating, respectively [2].

Insect antennae are primary organs for detection of olfactory cues. Antennae typically bear large numbers of sensilla, which house dendrites of a single or multiple olfactory receptor neuron(s) [1][3]. Sexual dimorphism is also evident in many species of insects, having differential antennal structure, sensillar distributions and sensillar morphologies. For example, Almaas and Mustaparta [4][5] reported that male tobacco budworm moth, *Heliothis virescens* F. (Lepidoptera: Noctuidae), possess long trichoid sensilla specialized for the detection of sex pheromones and short trichoid sensilla specialized for the detection of host plant odors; whereas females only have short, ‘generally tuned’, trichoid sensilla.

Dimorphism is also evident in the antennal lobe–the first synaptic output of ORNs from the antenna. The insect antennal lobe is a portion of the deutocerebrum of the insect brain and is usually demarcated by numerous spherical glomeruli (neuropil subcompartments) spread throughout which are the first synaptic neuropil of the olfactory system [6]. In many insects, there are a similar number of glomeruli in male and female insects, however, in most male moths pheromone-sensitive ORNs project axons to a sexually dimorphic macroglomerular complex (MGC)[6][7][8][9]. The MGC consists of several large glomeruli located closest to the base of the antennal nerve [10]. A varying number of satellite glomeruli surround a primary large glomerulus; *H*. *virescens* has four glomeruli in the MGC [6][7]. A similar, smaller, structure has been observed in female *H*. *virescens* at the base of the antennal nerve and is known as the medial and central large female glomeruli (mLFG and cLFG, respectively) [8]. We recently found that the cLFG receives significant innervation from olfactory receptor neurons which detect cis-9-tetradecenal (Z9-14:Ald), a primary component of *H*. *virescens* female sex pheromone [11][12].

In early studies, it was reported that male and female moths were both equally tuned to plant odors, but male moths were exclusively able to detect female pheromone components in the vast majority of cases [13]. However, Seabrook et al. [14] reported that some species of Lepidoptera do not show marked morphological sexual dimorphism, such as *Choristoneura spp*. Clemens (Lepidoptera: Tortricidae) and *Trichoplusia ni* Hübner, (Lepidoptera: Noctuidae) but can detect sex pheromone in relatively small concentrations. Ochieng et al. [15] found that *Spodoptera littoralis* Boisduval (Lepidoptera: Noctuidae) shows comparable ‘auto-detection’ of female sex pheromone relative to males. Electroantennograms (EAGs) of female *H*. *virescens* also show significant responses to components of conspecific female sex pheromone [11]. A large concentration of 'male' type pheromone binding proteins have been found in the antenna of female *H*. *virescens*; and female pheromone receptor gene expression is also present in female *H*. *virescens* antenna [16][17]. A study by Skiri et al. [18] using calcium imaging was unable to detect female pheromone activation in the antennal lobe of female *H*. *virescens*, however, it was reported that the cLFG was not in focus during the study. Conversely, Hillier et al. [11] reported that female *H*. *virescens* do indeed detect and respond to their own sex pheromone despite morphological sexual dimorphism of their trichoid sensilla; and that female pheromone-detecting ORNs do exclusively target the female cLFG analogous to the MGC in males. Despite this, the evolutionary and behavioral importance of the ability of female *H*. *virescens* to detect female pheromone is not yet known.

Biogenic amines are of particular importance in insect olfaction and are expressed in relatively few antennal lobe neurons [19][20]. Octopamine (4-(2-amino-1-hydroxy-ethyl) phenol; hereafter ‘OA’) is a neuromodulator of insect olfaction as well as a neurohormone and neurotransmitter throughout the nervous system [19][20]. The most widely, and arguably evolutionarily important, observation of OA is the apparent similarity with the noradrenergic system of vertebrates. In the periphery, OA functions as a regulator of activity and stress hormone, enhancing muscle contraction and increasing metabolism of the fat body [21][22].

All major areas of the insect brain (optic lobes, central body, and mushroom bodies) are innervated with octopamine containing neurons [22]. Octopaminergic neurons, as clusters of cell bodies and perikarya, are classified into ten groups (I-X) [23]. Of particular interest are the unpaired, Group VIII and IX, neurons situated in the ventral midline or the dorsal midline of the subesophageal, thoracic, and abdominal ganglia. These referred to as VUM (ventral unpaired median) and DUM (dorsal unpaired median) nerves and innervate most of the insect brain [23]. In the locust the DUMETi neuron (a DUM neuron) innervates the extensor tibiae muscle and modulates neuromuscular transmission; octopamine injections have been shown to increase the contractile force of this muscle [24]. In *Apis mellifera* L the VUMmx1 neuron originates in the suboesophageal ganglion and projects into the brain innervating the glomeruli of the antennal lobes, lateral protocerebrum and mushroom body calyces [25]. The VUMmx1 neuron has been proposed as being octopaminergic, possibly mediating olfactory learning, by providing reinforcement similar to that of sugar in olfactory learning trials [25][26].

The modulatory effect of OA on the olfactory system of insects is of particular interest. Within the Lepidoptera, octopaminergic neurons and OA receptors have been cloned from multiple species, with extensive evidence for its role in sensory processing and behavioral modulation [27][28][29]. In studies on oriental fruit moths (*Grapholita molesta* Busck), OA treatments cause a marked increase in sensitivity to pheromone signals resulting in increased numbers of males taking flight and orienting to the odor source in a wind tunnel [30]. Linn & Roelofs [31] also found male flight response to pheromone signals modulated by circadian rhythm, temperature and photoperiodic cues are linked to endogenous neuromodulators. Subsequent work with cabbage looper moths (*T*. *ni*) showed that not only were male moths sensitized to pheromone after OA injection but that their behavioral response was similar to response of untreated moths to much higher pheromone concentrations, and that OA antagonists reduced male response rates to female pheromone dramatically [32]. However, in studies with male gypsy moths (*Lymantria dispar* L. (Lepidoptera: Erebidae)) it was determined that the behavioral effect of OA injections did not occur at all times in the light cycle. Injections at the start of scotophase had no affect on pheromone response whereas injections only an hour earlier showed marked increase in pheromone response [33]. As well, in *Agrotis ispilon* Hufnagel (Lepidoptera: Noctuidae), it has been shown that OA treatment enhances flight of virgin males to female sex pheromone, it does not restore mating behavior in post-mated moths, despite restoring response characteristics within the antennal lobe [34]. These studies show that context is critical for the activity of OA. OA release, and its neuromodulatory action, is under circadian control and that light level detection is key in its regulation, and that mechanisms under OA control may be part of more complex regulatory pathways controlling behavioral outputs.

Centrally, increased intracellular cyclic adenosine monophosphate (cAMP) activity in the antennal lobe has been purported as a likely pathway for increased sensitivity to pheromone [35]. On the antenna, Pophof [36] also investigated transepithelial potential changes to observe the modulatory effects of OA on peripheral olfactory receptor neurons in the silkworm moth (*Antheraea polyphemus* Busck (Lepidoptera: Saturiniidae)). OA injections increased peak nerve impulse frequency in a dose dependent fashion in response to pheromone stimulation whereas the antagonist epinastine decreased sensitivity in a dose-dependent fashion [36]. It was concluded that the likely mechanism was modulation of the Na/K membrane pump of the ORNs via cAMP induced PKA action. These results were later confirmed in *Mamestra brassicae* L (Lepidoptera: Noctuidae) [37]. In addition they showed that chlorpromazine, an OA antagonist, decreases ORN pheromone sensitivity and may decrease long term adaptation of ORNs keeping them in a sensitized state despite long exposure to pheromone cascade [37]. OA injections also increase ORN responses to pheromones in male *Bombyx mori* L. (Lepidoptera: Bombycidae) but did not increase ORN responses to general odorants in females [38]. This led to the conclusion that despite the olfactory transduction pathway being the same for pheromone-sensitive and general odorant-sensitive ORNs a modulatory pathway exists in male pheromone sensitive sensilla that is absent or less sensitive in females.

Recent evidence found that a large portion of female *H*. *virescens* short trichoid sensilla do detect their conspecific female sex pheromone, which lends the question as to whether these sensilla are modulated by OA [11]. OA is expressed on the antennae of *H*. *virescens*, with reactive cells present at the base of sensilla [17]. Given this knowledge, does OA also increase the sensitivity of these sensilla, making them similar to the long pheromone sensitive trichoid sensilla of male *H*. *virescens*? Or is their sensitivity not increased by OA, making them pharmacologically similar to other short trichoid sensilla tuned to non-pheromone odorants? Based on the evidence of octopaminergic neurons in the brain and receptors in the antenna of female *H*. *virescens* it is hypothesized that OA will increase the sensitivity of female ORNs to their conspecific female sex pheromone. In effect, this would indicate a significant degree of similarity in neuropharmacology between male and female pheromone detecting systems, despite morphological differences. The objective of this study is to utilize single sensillum recordings (SSR) to compare any modulatory effect of OA injections on male and female *H*. *virescens* response to pheromones and host plant volatiles.

## Materials and Methods

### Insects

Three to four day old *H*. *virescens* were selected from an established colony at Acadia University (Wolfville, Nova Scotia). Larvae were reared on Tobacco Budworm Diet purchased from Southland Products, Inc. (Southland™ Products Inc., Lake Village, Arkansas). Pupae were then separated by sex and placed into an environmentally controlled room (24°C, 60% relative humidity) on a reversed light schedule (14D:10L) until eclosion and testing.

### Chemicals

Female pheromone components were selected based on their ability to elicit single sensillum responses in female *H*. *virescens* moths [11]. The female sex pheromone components were as follows: (Z)-9-tetradecenal (Z9-14:Ald), (Z)-11-hexadecenal (Z11-16:Ald) and (Z)-11-hexadecenyl acetate (Z11-16:OAc) and were obtained from Bedoukian Research, Inc. (Danbury, Connecticut, USA). Putative host volatiles were selected from compounds that have shown physiological or behavioral effects on *H*. *virescens* in previous studies [11][12][39][40]: 2-phenyl ethanol, Z3-hexenol, along with racemic linalool and β-caryophyllene were obtained from Sigma Aldrich (St. Louis, MO, USA). All chemicals were diluted in hexane at decade steps from 10ng/μl-100μg/μl, and stored at -20°C. OA-hydrochloride was obtained from Sigma Aldrich (St. Louis, MO, USA) and stored at room temperature. OA was diluted to 50μg/μl in hemolymph Ringer solution [41] and stored in a fridge at 4°C.

### Odorant Stimulation

Z11-hexadecenal (Z11-16:Ald), Z9-tetradecenal (Z9-14:Ald) and Z11-hexadecenyl acetate (Z11-16:OAc) were used as pheromone stimuli, whilst 2-phenyl ethanol, Z3-hexenol, (+/-) linalool and β-caryophyllene were selected as host volatiles for testing. All stimuli were diluted to 10^−4^ to 10^2^ μg/μl, applied to filter paper at 10^−3^ to 10^3^ μg loads and inserted into glass pipettes for stimulations. Hexane was used as the solvent blank for control stimulations. Stimuli were introduced as a 100ms pulse to a continuous stream of humidified, charcoal filtered air at a flow rate of 1 L/min. When a sensillum was contacted and a stable recording achieved, each stimulus was presented in random order at a 1μg stimulus load, along with the hexane blank to determine relative sensitivity to all compounds. After an observed response to an individual odor stimulus, a full concentration series was tested.

An interval of at least 60 seconds was allowed to pass between stimulation to prevent adaptation, in addition a constant vacuum flow (30 cm/s) was provided to clear residual odor away from the preparation between stimulation. Stimulations were carried out for 100ms and recordings were initiated 2 seconds before stimulation and continued 4 seconds after stimulation for a 6 second total recording. All recording were made using Spikehound software (Physiology Recording and Identification of Multiple Events; MATLAB®, The Mathworks, Inc.)[42], a freely-available Windows based program for digital recording and analyses of event/spikes.

### OA Injections

All moths were individually restrained in cut disposable 1ml micropipette tips with dental wax. Injections of OA (1 μl volume) were made using a 50 μl syringe at a 1mm depth to the vertex (posterior head, ‘neck region’), corresponding to 0.1 μg OA/mg of body weight per moth [38]. SSR were conducted 30 minutes after injection to coincide with previous reports of the optimal window of activity of OA on ORNs [38][39]. Control moths were handled in a similar manner, but injected with Ringer only.

### Single Sensillum Recordings

Restrained, injected moths were placed horizontally on a microscope slide with their antennae fastened to a small plastic cube with water-soluble correction fluid. Correction fluid was also applied to the base of the antenna to ensure stability. The reference electrode was inserted in the ipsilateral eye. The preparation was viewed at 200x magnification under a Nikon Eclipse Fixed Stage Microscope.

Glass electrodes were produced using borosilicate capillary glass in a Sutter Instrument Co P-97 Flaming/Brown Micropipette Puller (Sutter Instruments, Novato, CA). Individual sensilla were cut using fine glass capillary mounted on a piezoelectric crystal controlled by a function generator (INSTEK GFG-8215A). The glass capillary was made to resonate at high speed by altering the frequency (square wave and 100K signal amplification setting) of the signal from the function generator to the sound emitting piezo crystal [11][43]. Advancement of the resonating glass capillary with the micromanipulator allowed the distal 25% of a given sensillum selected from the proximal ventral portion of the antenna to be cut.

Once a sensillum was cut, a glass-recording electrode (chloridized silver wire in a Ringer -filled glass capillary) was micromanipulated to the tip of the cut sensillum. ORN activity was filtered and amplified using an EX-1 amplifier (Dagan Corporation, Danbury, CT, USA; Low Cut Filter:10, High Cut Filter: 3K, Gain: 50, Notch Filtered), monitored using a Oscilloscope (GOS-620FG, Instek, New Taipei City, Taiwan) and converted to audio using a AM Systems, Inc. 3300 Audio Monitor for ease of ORN activity detection. Signals digitally acquired and passed to a computer via a DAQ card (PCI-6251; National Instruments, Austin, TX, USA). As mentioned previously odor stimulations and subsequent responses were controlled and digitally recorded using Spikehound® software.

### Data Analysis

Recordings were filtered (300 high cut, 3000 low cut) to remove any background electrical noise. Spikehound was used to extract, analyze and save the nerve impulse (i.e. spike) data. Individual ORNS were sorted by spike amplitude. Stimulus-induced spike frequencies were calculated using a one second window following stimulus onset. Pre-stimulus spontaneous impulse frequencies were calculated from a 1 second window during the recording (time points 0–1 second before stimulus delivery. Pre-stimulus and post-stimulus data were exported as text files.

The mean change in impulse frequency (post-stimulus frequency–pre-stimulus frequency) for all odors was calculated for the control and OA injected groups across the concentration series, and standardized versus a solvent (hexane) control stimulus. Data was segregated by sex, and a multi-way ANOVA was performed to testing injection treatment, stimulus and concentration as factors [44]. Mean pre-stimulus spike frequency was also calculated to compare spontaneous firing prior to stimulus onset. A two-way ANOVA was conducted comparing pooled spontaneous activity of pheromone-sensitive versus host volatile sensitive sensilla, and injection of OA or control factors. Tukey's HSD (honest significant difference; **α** = 0.05) test at was used for post-hoc comparisons. All statistical analyses were conducted in in JMP® 10 [45].

## Results

Successful recordings (complete recordings in which responses to all stimuli were obtained) were made from 239/400 sensillar contacts from females, and from 275/365 contacts in males in the control group (the greater success rate in males is attributed to ease in identifying pheromone-sensitive long sensilla trichodea; Table 1). In the OA-injected group, 119/212 sensillar contacts yielded useful recordings from females, along with 140/189 contacts in males.

**Table 1:** Number of successful single sensillum recordings from male and female Heliothis virescens injected with ringer or octopamine which responded to selected stimuli.

	Female		Male	
Successful recordings	Ringer	Octopamine	Ringer	Octopamine
2-Phenyl ethanol	15	15	15	15
Beta-caryophyllene	25	20	21	20
Linalool	20	24	20	24
Z3-hexenol	15	15	15	15
Z9-14:Ald	15	15	23	22
Z11-16:Ald	15	15	21	20
Z11-16:OAc	15	15	20	24
**Total**	**120**	**119**	**135**	**140**

**Fig 1:**
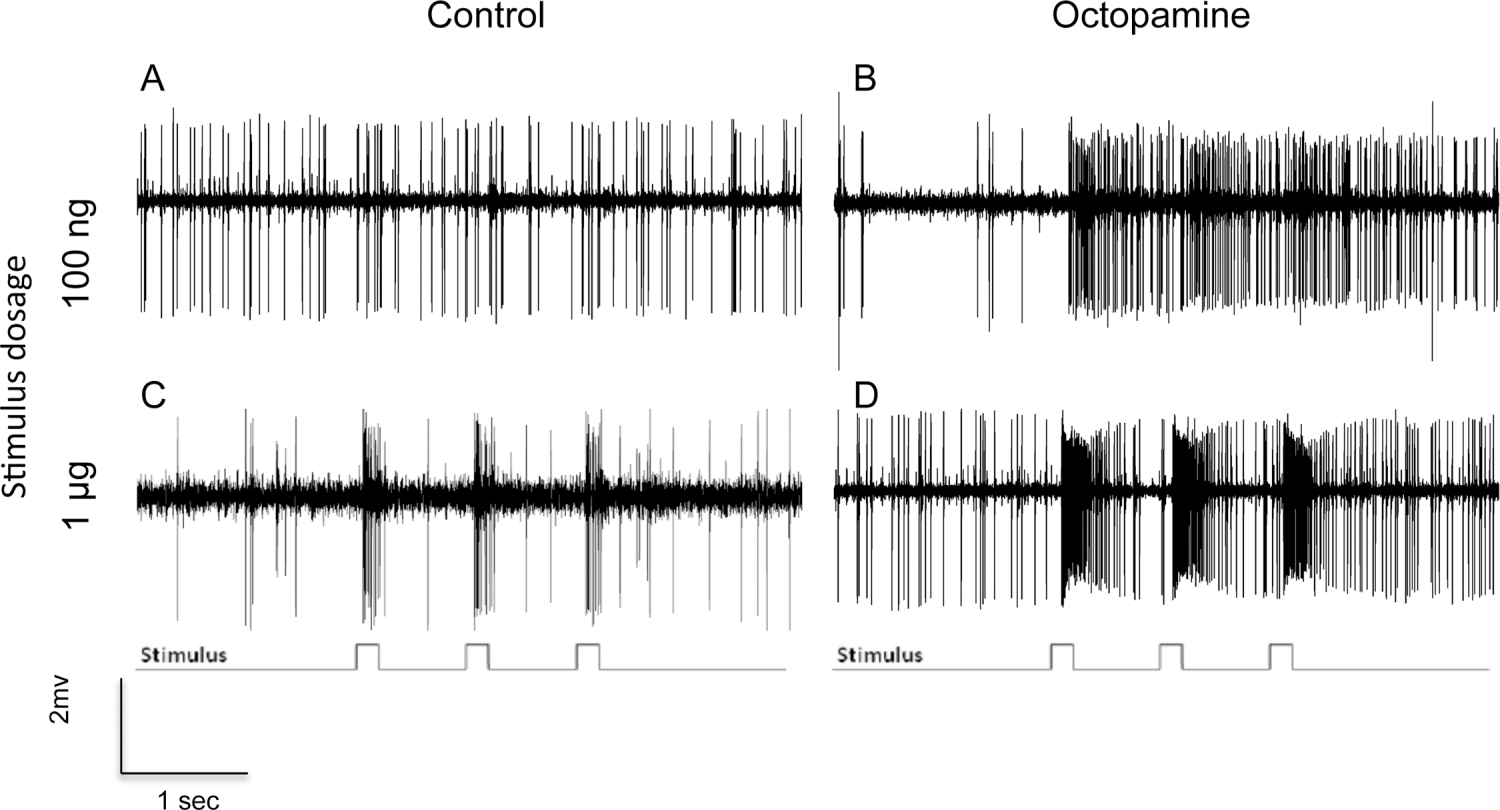
Exemplar responses of female type 3 sensilla to stimulation of three 100ms pulses of Z9-14:Ald at concentrations of 100ng and 1μg following injection of Ringer control or octopamine.

**Fig 2:**
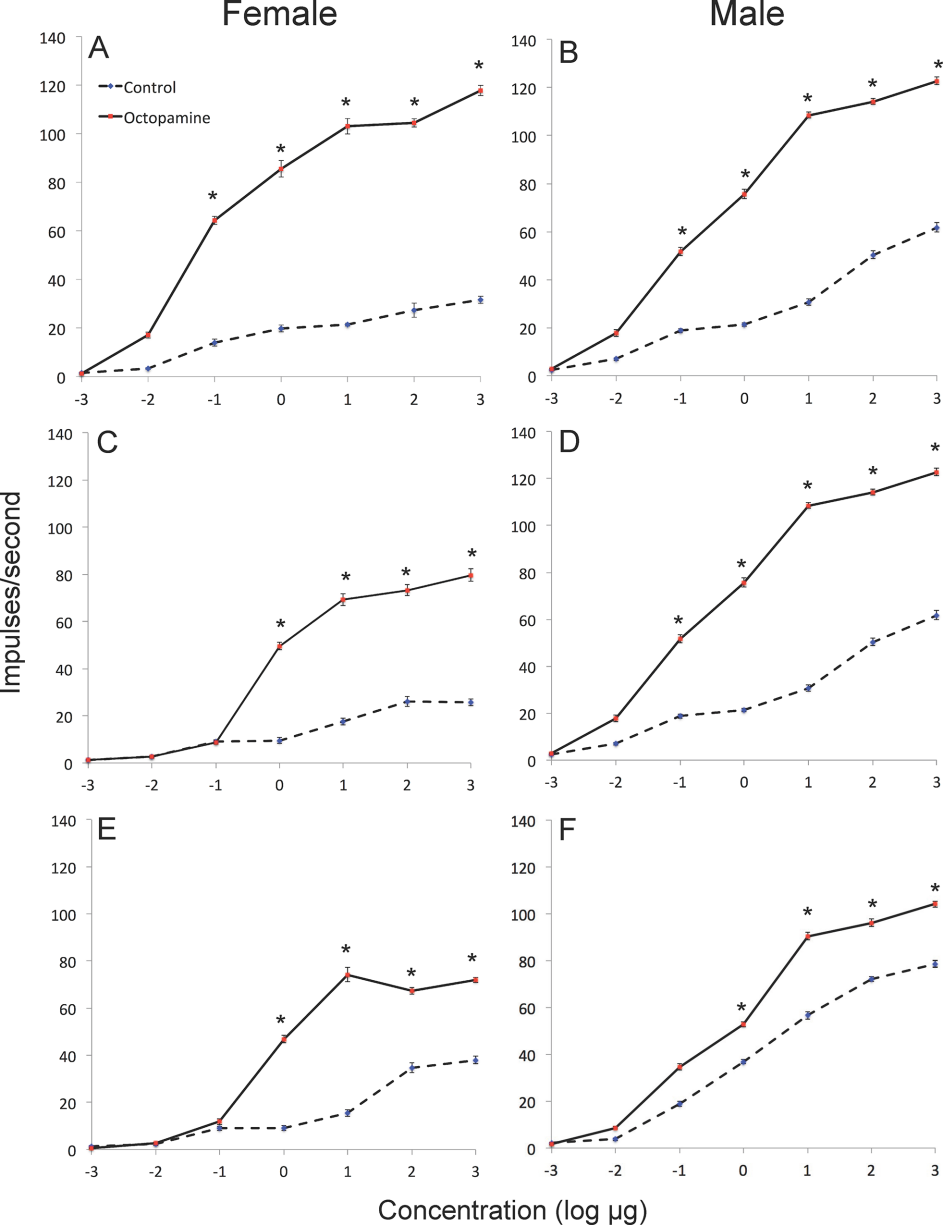
Dose-response curves showing impulses/second from sensillar recordings of male and female *Heliothis virescens*, following injection of Ringer control or octopamine, and stimulation with: (A, B) Z9-14:Ald; (C, D) Z11-16:Ald; and (E, F) Z11-16:OAc. Asterisks indicate significant differences between responses in control and octopamine treatments to the same compound/concentration of stimulus (Tukey’s HSD, p<0.05).

Mean spontaneous impulse frequency of ORNs within pheromone-sensitive sensilla was significantly greater in the OA-injected group than in the control group for both males (F_3, 155_ = 107.2; p<0.001) and females (F_3, 194_ = 97.5; p<0.001). In males, responses by ORNs housed in long sensilla trichodea were represented by Type A, responding to Z11-16:Ald, Type B, responding to Z9-14:Ald, and Type C responding to Z11-16:OAc, and other components. In females, responses to selected pheromone components were localized to short sensilla trichodea which have been previously shown to respond to key pheromone components. Pheromone component-sensillar types were as follows: Z11-16:Ald (Type 5), Z9-14:Ald (Type 3), Z11-16:OAc (Type 1). Nomenclature for sensillar types for males and females is based on numerous previous studies (4)(5)(11)(34). ORNs from OA-injected female (F_1,90_ = 4853.2; p<0.001) and male (F_1,130_ = 5757.4; p<0.001) moths were significantly more sensitive to all female sex pheromone compounds than control moths (Figs 1 and 2). This was observed in recordings from ORNs housed in long sensilla trichodea in males and from those within short sensilla trichodea in females, which responded to these stimuli. This effect was most pronounced at stimulus concentrations above 100ng/μl-1μg/μl.

**Fig 3:**
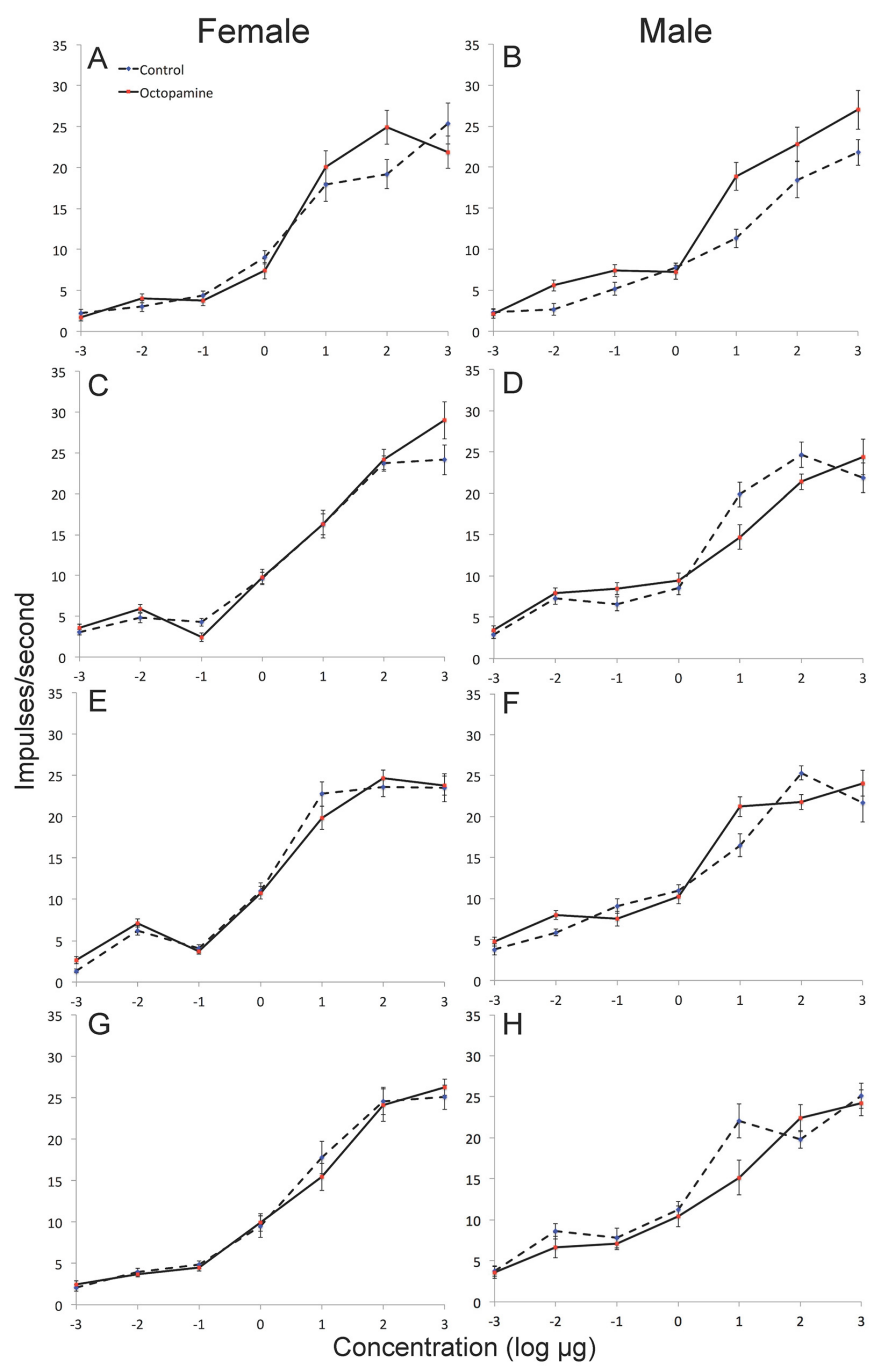
Dose-response curves showing impulses/second from sensillar recordings of male and female *Heliothis virescens*, following injection of Ringer control or octopamine, and stimulation with: (A, B) 2-Phenyl ethanol; (C, D) β-caryophyllene (E, F) linalool; and (G, H) Z3-hexenol. No significant differences were noted between responses in control and octopamine treatments to the same compound/concentration of stimulus (Tukey’s HSD, p<0.05).

By comparison, no significant differences were observed in ORN responses within short sensilla trichodea between control and OA-injected groups of both sexes when stimulated with selected host plant stimuli (females—F_3,149_ = 0.5727; p = 0.45; males—F_3,145_ = 2.5; p = 0.11; Type 7–13 sensilla in both males and females; Fig 3). Increasing stimulus concentrations within both sexes and experimental groups yielded very similar stimulus profiles (Fig 3). Furthermore, the spontaneous impulse frequency of host volatile-sensitive neurons in both sexes was not significantly different following injection of OA (F_2,294_ = 0.49; p = 0.65).

## Discussion

Single sensillum recordings compared the response of *Heliothis virescens* pheromone-sensitive sensilla (long sensilla trichodea in males and short sensilla trichodea in females), and those detecting a selection of host plant volatiles (short sensilla trichodea in both sexes). OA injections selectively increased sensitivity of male and female *H*. *virescens* olfactory receptor neurons to pheromone components versus other compounds tested. This study further confirms previous work by Hillier et al. [11] that *H*. *virescens* females can detect components of their own sex pheromone.

Following OA injection, threshold to response by males and females to pheromone components was reduced by two-to-three orders of magnitude versus control group. In other words, OA treatment confers a sensitivity and response to a 100ng/μl stimulus which is as great as the response to a 10μg/μl simulus in a control moth (three orders of magnitude greater). In addition, an increase in spontaneous firing was observed in pheromone-sensitive ORNs from both sexes, but not to those receptive to the host volatiles.

The mechanism of OA action on neurons is proposed via the G-coupling of OA receptors to adenylate cyclase [35]. Although the underlying mechanisms of transduction are not understood, it has been proposed that OA binding induces the production of cAMP, and OA-dependent CAMP levels putatively regulate pheromone receptor ion channel complexes, modifying sensitivity to key odorants [46]. Subsequent increases of subthreshold potentials (and sensitivity) have been proposed through increased leaking of the olfactory receptor/coreceptor ion channel complex [47][48], and/or increased opening of PKC-inhibited L-type Ca 2+ and CNG-channels [47][49]. Overall, the current work suggests biogenic amine susceptibility, and thus OA receptors, may be linked to olfactory receptor protein types which are differentially expressed in both long and short sensilla trichodea in male and female moths.

As discussed, until relatively recently, much insect olfactory research has been based on the presumption that many species of female moths may not effectively detect female sex pheromone. The combination of morphological and physiological data to date has therefore suggested that dimorphism in the detection and processing of pheromones maintains exclusivity based upon sex. Female *H*. *virescens* do, however, have sensilla that detect, and a cluster of glomeruli at the base of the antennal lobe which process, female sex pheromone components [11]. This study further validates that females both detect and modulate pheromone responses in a manner similar to conspecific males.

Modulation of ORN sensitivity in male moths is not surprising. It is logical that increasing sensitivity to female sex pheromone is extremely important to male moths. Indeed, Roelofs and Linn [50] demonstrated that injections of OA cause male moths to detect, take flight, and orient to smaller concentrations of female pheromone in wind tunnel assays. However, minimal behavioral research has been conducted as to why females detect their own sex pheromone. Indeed, few reliable behavioral paradigms have been devised to examine if females respond behaviorally to female sex pheromone, while many exist exquisitely showing that males orient and fly upwind to female pheromone sources [51][52]. As a consequence, more research is required to determine if increased peripheral sensitivity to pheromone in females will affect behavioral response to pheromones.

While sensitivity of female ORNs to pheromone components has been previously documented [15], the selective modulation of such ORNs in a manner consistent with male pheromone-sensitive ORNs is novel. Such modulation is proposed to impact yet undescribed pheromone-mediated behaviors in females for which modulation of sensitivity is required (similar to males [30][31][32][33]). As in males, this may influence circadian rhythms of response, orientation, or calling behavior directly [53][54][55][56]. Without a clear understanding of the role of such ‘autodetection’, it remains to be determined how OA modulation of peripheral sensitivity influences olfactory-driven behaviors or physiological changes.

Three primary hypotheses exist for female ‘autodetection’: 1) First, female moths may detect the pheromone mixture produced by other conspecific females, and thus, can position themselves to reduce intraspecific competition for mates [53]. Second, pheromone emission may trigger females to join together to form choruses of joint pheromone-emission [54]. Third, the detection of conspecific female pheromone by immature female moths triggers a biochemical process (i.e. priming) by which immature female moths initiate production and emission of their own sex pheromone [55][56]. It is clear, however, more research is required in this area, and no previous evidence is known to support either hypothesis specifically for female autodetection in *H*. *virescens*.

Recent electrophysiological evidence has shown that females of two Noctuid moth species, *S*. *littoralis* and *H*. *virescens*, detect their own female sex pheromone [11][15] despite having only morphologically ‘short’ trichoid sensilla. Additionally, olfactory receptor neurons of these female sex pheromone-detecting sensilla target a large glomerulus (central large female glomerulus, aka. cLFG) at the base of the antennal nerve analogous to the MGC in female *H*. *virescens* [11]. As well, female pheromone receptor gene expression and 'male' type olfactory binding proteins have been previously found on *H*. *virescens* antenna [16][17]. From an evolutionary perspective, our study demonstrates octopaminergic effects on ORNs within female *H*. *virescens* pheromone-sensitive sensilla that suggests similarity in their physiological function as ORNs in male pheromone-sensitive sensilla. This suggests that pharmacological specialization of pheromone-sensitive ORNs via octopaminergic modulation predates divergence of sexually dimorphic morphological differences seen in this species (i.e. differences in sensillar length and a relatively large MGC).

Pophof [38] reported that OA injections in males increased their sensitivity to female sex pheromone, but similar injections in females did not increase sensitivity to general odorants. This suggests that an OA-dependent modulatory pathway is either absent, or less sensitive, in female ORNs. It should be noted that to date, conspecific female pheromone-detecting ORNs in female *A*. *polyphemus* have not been reported [38].

It is worthwhile noting that other studies provide evidence of OA-modulation of peripheral olfactory responses to non-pheromonal odors. In Tortricid moths *Choristoneura rosaceana* Harris and *Arygrotaenia velutinana* Walker, injection of OA enhances electroantennogram responses to several host plant volatiles, mimicking sensitization observed by pre-exposure to complex host mixtures [57]. As well, cockroaches, *Periplaneta americana* L, have differential modulation of olfactory receptor neurons. Specifically, application of OA to male cockroaches increased ORN responses to the non-pheromone volatile (hexan-1-ol) [58]. This study further found that different sensilla responsive to non-pheromone volatiles were influenced differently, with firing rates of short, but not long-alcohol-sensitive sensilla being increased. Differential results between results found in cockroaches and other studies on Lepidoptera remain to be reconciled, however, each of these studies used differing concentrations of OA injection, and results garnered from other studies on Lepidoptera using electroantennograms may include responses from many different types of sensilla (comprising both pheromone and host-plant sensitive sensilla).

Recently octopamine/tyramine receptors have been cloned from a *Mamestra brassicae* L., with extensive expression noted within both long and short sensilla trichodea of both sexes [59], also suggesting that OA modulates allelochemical and pheromone detection. This varies from previous Digoxigenin (DIG)-labeling results which found OA expression in *H*. *virescens* antennae was isolated to cells at the base of sensilla, rather than within sensilla themselves [29]. Therefore, behavioral and evolutionary significance of the set of odorants tested in each species, along with the context of presentation and endogenous OA concentrations may also influence observed responses.

Evidence that OA enhancement of ORN sensitivity in *H*. *virescens* is exclusive to pheromone-sensitive neurons (at least based on the set of odorants tested) provides new directions for investigation of OA on non-pheromone based behaviors. OA can replace the sugar stimulus in honey bee proboscis extension reflex (PER) conditioning trials, and concurrent OA injection can greatly increases PER responsiveness to sucrose [60][61]. More recently, work on *Manduca sexta* L. has also demonstrated a marked effect of OA injection on conditioning to floral volatile organic compounds, displaying PER responsiveness to OA, and multichannel neural recordings which were similar to those obtained from moths which had learned with a standard PER protocol [62]. Future work in *H*. *virescens* might therefore investigate if OA exhibits effects on olfactory conditioning using odorants documented in the current study, and if these may be modulated through higher order activity in the brain, rather than via direct enhancement of peripheral sensitivity.

In conclusion, this study demonstrates that OA dramatically increases the sensitivity of female insect olfactory receptor neurons, specifically those detecting female-produced sex pheromone components. This provides new avenues to examine the connection between the modulatory role of OA on pheromone-detecting ORNs and female moth behavior. Moreover, these results provide new insights regarding the divergence and evolution of sexual dimorphism.

## Abbreviations

cLFG: Central large female glomerulus
cAMP: cyclic adenosine monophosphate
mLFG: medial large female glomerulus
OA: Octopamine: OA
ORN: olfactory receptor neuron
MGC: macroglomerular complex
SSR: single sensillum recording
PKA: protein kinase A
PER: proboscis extension reflex
Z9-14:Ald: cis-9- tetradecenal
Z11-16:Ald: cis-11-hexadecenal
Z11-16:OAc.: cis-11-hexadecenyl acetate: Z11-16:OAc
